# The Mechanisms of Movement Control and Time Estimation in Cervical Dystonia Patients

**DOI:** 10.1155/2013/908741

**Published:** 2013-10-01

**Authors:** Pavel Filip, Ovidiu V. Lungu, Daniel J. Shaw, Tomas Kasparek, Martin Bareš

**Affiliations:** ^1^Central European Institute of Technology, CEITEC MU, Behavioral and Social Neuroscience Research Group, Masaryk University, 625 00 Brno, Czech Republic; ^2^First Department of Neurology, Faculty of Medicine, Masaryk University and St. Anne's Teaching Hospital, 656 91 Brno, Czech Republic; ^3^Department of Psychiatry, University of Montréal, Montréal, QC, Canada H3C 3T5; ^4^Functional Neuroimaging Unit, Research Center of the Geriatric Institute Affiliated with the University of Montréal, Montréal, QC, Canada H3C 3T5; ^5^Department of Research, Donald Berman Maimonides Geriatric Center, Montréal, QC, Canada H3C 3T5; ^6^Department of Psychiatry, Faculty of Medicine, Masaryk University and St. Teaching Hospital, 625 00 Brno, Czech Republic

## Abstract

Traditionally, the pathophysiology of cervical dystonia has been regarded mainly in relation to neurochemical abnormities in the basal ganglia. Recently, however, substantial evidence has emerged for cerebellar involvement. While the absence of neurological “cerebellar signs” in most dystonia patients may be considered at least provoking, there are more subtle indications of cerebellar dysfunction in complex, demanding tasks. Specifically, given the role of the cerebellum in the neural representation of time, in the millisecond range, dysfunction to this structure is considered to be of greater importance than dysfunction of the basal ganglia. In the current study, we investigated the performance of cervical dystonia patients on a computer task known to engage the cerebellum, namely, the interception of a moving target with changing parameters (speed, acceleration, and angle) with a simple response (pushing a button). The cervical dystonia patients achieved significantly worse results than a sample of healthy controls. Our results suggest that the cervical dystonia patients are impaired at integrating incoming visual information with motor responses during the prediction of upcoming actions, an impairment we interpret as evidence of cerebellar dysfunction.

## 1. Introduction

Cervical dystonia, the most frequent adult focal dystonia, is a syndrome characterized by involuntary twisting movements of the head, leading ultimately to temporary or constant abnormal postures interfering with voluntary movement [1, 2]. Despite over a century of research since its first description in the literature [3], the pathophysiology of this disease still remains elusive. Aberrant activity in the basal ganglia has been noted repeatedly as the main cause of the sustained cocontraction of opposing agonist and antagonist muscles [2, 4, 5]. Recently, however, the cerebellum—first noted in dystonia pathophysiology over 25 years ago [6]—has received considerable attention [7–9]. Neurophysiological [10] and neuroimaging studies [11], showing increase in gray matter density in cerebellum [12], abnormal cerebellar activation in various tasks [13–15], and increased glucose metabolism in cerebellum [16, 17], clearly demonstrate its involvement in dystonia. Furthermore, an elegant review of 25 secondary cervical dystonia cases connects the pathophysiology of cervical dystonia primarily with cerebellar lesions [18]. 

For a long time the cerebellum has been associated exclusively with motor functions. Increasingly, though, the cerebellum is implicated in a wide spectrum of different process controls extending far beyond the typical cerebellar domain. These include, for example, attention [19, 20], associative learning [21], motivation control [22], and, most relevant to the present study, time assessment [23–26]. The precise role of the cerebellum in the representation of time and a delineation of basal ganglia function in this respect are still a matter of continuous research [27–29]. To date, there is evidence of two dissociable neural timing systems: an “automatic” system, involving the cerebellum and linked closely to motor networks, is responsible for discrete event timing in the range of milliseconds [28, 30, 31]; a “cognitively controlled” system, comprised of basal ganglia and cortical structures focused on attention and memory requirements, deals instead with events in the range of seconds [29, 32]. 

In accordance with this distinction, we have shown in previous studies that subjects with severe cerebellar damage [23] or less severe dysfunction of the cerebellum [25] have poorer performance on tasks requiring motor timing at the sub-second level. Interestingly, on the very same task, patients with early stages of Parkinson's disease (PD) did not differ significantly from healthy controls [25]. Since PD is associated strongly with basal ganglia dysfunction, this is consistent with the above-mentioned distinction and other studies focusing on timing in PD [33, 34]. The current study builds on this research by investigating the performance of cervical dystonia patients on this motor-timing task. On the basis of the aforementioned evidence, we assumed some cerebellar dysfunction in our sample of cervical dystonia patients. We hypothesized, therefore, that these patients would perform poorly at this task relative to a healthy control group due to disrupted time estimation in very short intervals.

## 2. Methods and Materials

### 2.1. Participants

Thirty healthy individuals (15 males; mean age = 55.5 yrs, SD = 12.6 yrs) and 30 primary cervical dystonia (CD) patients (14 males; mean age = 52.0 yrs, SD = 13.65 yrs) participated in the study. All patients showed only symptoms of pure cervical dystonia—there were no further dystonia signs (for more information see Table 1). The cervical dystonia subjects did not suffer from hand tremor nor abnormal upper-arm posture. Only patients with no shoulder elevation or a slightly elevated shoulder (maximally moderate elevation with 1/3–2/3 possible movement range) participated in the study. All subjects were right-handed. None of the subjects had history of color blindness. According to the Montgomery and Asberg Depression Rating Scale (MADRS), no subjects suffered from clinical depression (mean score = 6.50, SD = 6.85) [35]. Prior to testing, the patients were scored on the Toronto Western Spasmodic Torticollis Rating Scale (mean score = 10.2, SD = 4.64) [36]. The patients were recruited from the Movement Disorders Outpatient Clinic at St. Anne's University Hospital, Brno, Czech Republic. The study was approved by the hospital's Institutional Review Board.

### 2.2. The Task

**Figure 1:**
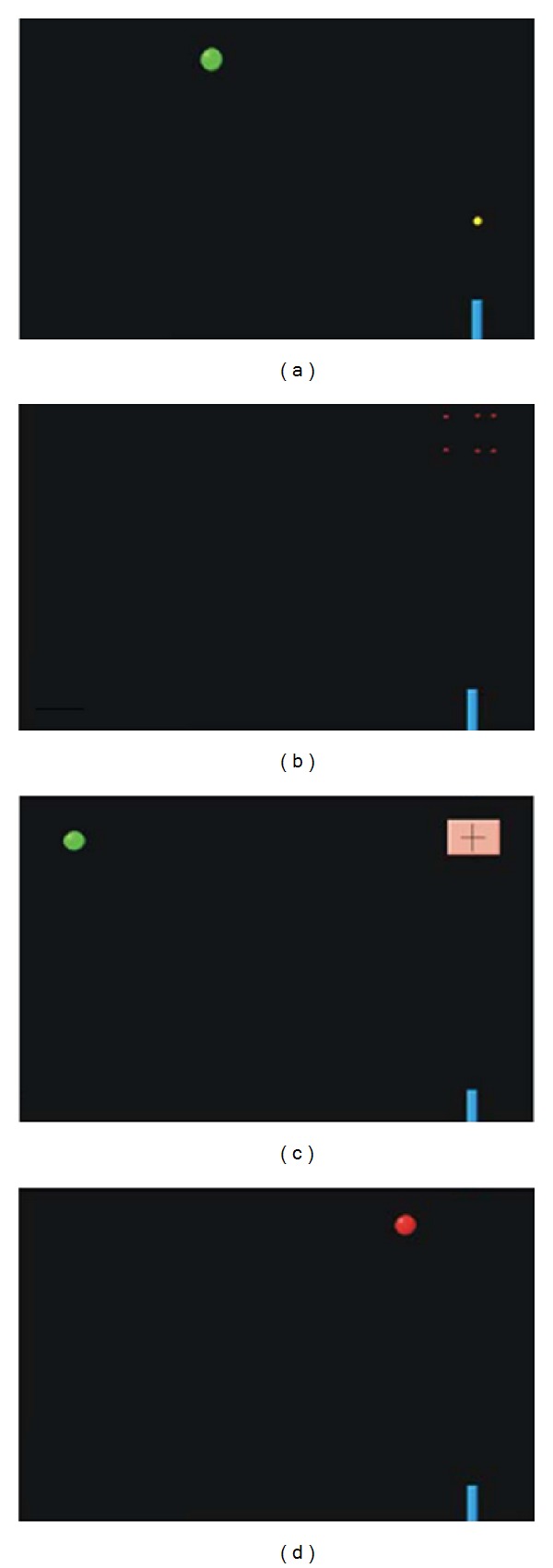
The task and experimental conditions. (a) The main interception task. The green ball moves from the left side of the screen to the interception zone in the upper right corner of the screen (as for the position, corresponding to the pink cross square in Figure 1(c)). The blue “gun” in the lower right corner fires a “projectile” travelling at a constant speed to intercept the green ball. (b) Successful hit. If the ball is intercepted by the projectile, both objects explode. In case of miss, there is no animation. (c) Control condition 1. There is no projectile in this condition. The subject is supposed to press the button at the very moment the green ball reaches the interception zone marked by the cross inside the pink square. If the subject is successful, the ball explodes. (d) Control condition 2: the green ball once again moves from the left side of the screen to the interception area. However, there is no gun/projectile or pink interception square. The subject is supposed to press the button as soon as the ball changes its color from green to red.

The subjects performed the same motor-timing computer task as that employed in our earlier studies [23, 25]. In this task, participants are required to press a key with the dominant hand in order to launch a “projectile” that will intercept a circular green “target” object moving from the left side of the screen toward the upper-right corner (Figure 1). This projectile is launched from the lower-right corner of the screen and travels at a constant speed of 20.0 cm/s on an upward and unchanging trajectory. Participants are instructed to launch the projectile at the optimal time for it to hit the moving target in a prespecified, stationary, and fixed interception zone, positioned in the upper-right corner of the screen (Figure 1(a)). Following a successful interception, or “hit,” a small explosion animation is produced as a feedback for the subject (Figure 1(b)). In case of failure, no explosion occurs. 

On each trial, the green target is launched from the left edge of the screen and travelled towards the fixed interception zone at three possible angles (0°, 15°, and 30°) relative to the horizontal plane of the screen. It travels in three different manners (i.e., constant velocity, deceleration, and acceleration) and at three different speeds (slow, medium, and fast). Ergo, with all possible combinations of these variables (type, speed, and angle), the target can travel in 27 different ways. The green target diameter is 1 cm; the projectile diameter is 0.3 cm. Trials were organized into 12 blocks, each with a pseudorandomized combination of target movement parameters (type, speed, and angle). Each particular movement combination was presented twice on each block, with each block formed by 54 trials (27 combinations × 2 instances). Therefore, each movement combination occurred 24 times (12 blocks × 2 instances) during the whole procedure. The blocks were separated by breaks of 20-second durations. The entire procedure lasted approximately 35 minutes. Before the task, subjects underwent one experimental block as practice. When performing the task, participants were seated 60 cm in front of the 14′′ computer screen. No special amendments or mechanical supports for the head and arms were used. Subjects feeling discomfort or pain during the task were excluded from the final analysis.

In addition to the experimental condition described above, subjects performed two control conditions. The first (control condition 1 [CC1]) involved instantaneous interception of the moving target comparable to the experimental condition. Participants were required to press the button as soon as the target reached the interception zone, which was marked by a pink crosshair (Figure 1(c)). In this case, subjects were not required to estimate the travel time of the projectile; in other words, this condition tested the ability of the subjects to judge the temporal characteristics of the target under less demanding circumstances. The second control condition (CC2) involved the detection of the target color change. Here, the participants were required to press the button as quickly as possible when the moving target changed color from green to red (Figure 1(d)). The color change occurred randomly along its trajectory. In this condition, we tested simple reaction time that required accurate pursuit of the target along its trajectory but no estimation of target movement. The subjects were shown explicitly the transition from green and red targets in advance and asked if they were able to detect accurately the color change.

### 2.3. Data Analysis

The variables of interest were the hit ratio (i.e., the number of successful hits relative to the total number of trials) across the different movement combinations (i.e., speed, angle, and type), the total number of hits and early and late errors (i.e., subjects pressing the button before or after the target entered the interception zone), and the response time (RT). In order to use parametric statistical techniques, requiring a normal distribution for the hit ratio (normally a binary variable in each trial), we computed the percentage of hits for each subject and for each trial type in each block, and we then averaged these values across blocks. When comparing the number of hits and early and late errors, we employed nonparametric techniques; namely, we employed the Chi-square test to compare the distribution of early and late errors between the control group and the patient group. Given that the outcome of an individual trial (hit, early error, and late error) may be influenced by the outcome obtained in the previous trial, we performed a trial-by-trial analysis to determine the extent to which subjects were able to use their very last experience (the previous trial) to improve their performance in the current trial. In this case, we used the Chi-square test and the Cramer's *V* and phi coefficient (a correction of Chi-square as a function of the number of cases considered).

The task was designed to produce varying levels of difficulty. Specifically, the individual combinations of variables lead to different time windows where a successful interception was possible (in the range of about 50 to 175 ms); the shorter the time interval to press the button, the more difficult the task; for example, higher target speeds were more difficult to intercept. Therefore, we expected higher hit ratios for trials with wider compared with shorter time windows. The effects of the movement parameter combinations on the hit ratio were assessed using a general linear model analysis (GLM). Finally, we compared the hit ratio between blocks 1–6 and blocks 7–12 to evaluate possible learning effects during the course of the experiment.

All analyses were performed in SPSS (SPSS Inc., Chicago, IL, USA).

## 3. Results

In the following analyses, we have excluded the “angle” movement parameter since it had been proven to be of no significant effect on hit ratio or reaction time [23, 25]. We did not notice any fatigue of motor or cognitive nature in the subjects.

### 3.1. Reaction Time

**Figure 2:**
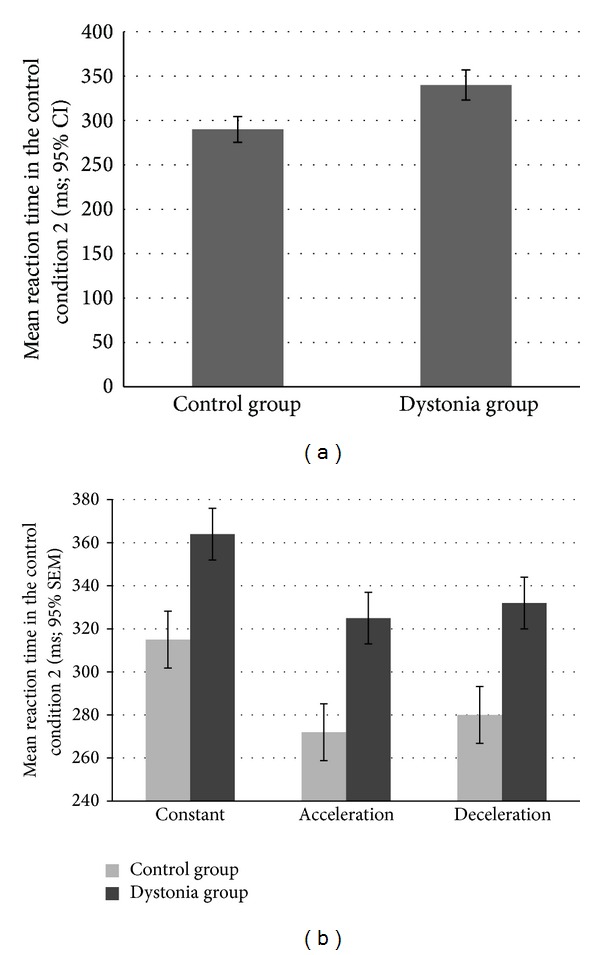
Reaction times. (a) Mean reaction time in the control condition 2: the reaction time of the patients was significantly slower than the reaction time in the control group. (b) Mean reaction times as a function of movement type in two groups: These results show that even if the patient group was generally slower (Figure 2), the reaction time was not affected by other parameters of the moving target (speed, acceleration) in a different way. This finding excludes the eventuality that the differences between the groups in the other main parameters may be due to oculomotor difficulties in the cervical dystonia group [37].

In the control condition CC2, we found a significant difference in the reaction time to color change between the patient group (mean = 341.84 ms, SD = 57.40) and the healthy group (mean = 288.72 ms, SD = 32.10 [*F*_1,58_ = 19.38; *P* < 0.0001]). As shown in Figure 2(a), this revealed that healthy controls were faster in the task than the patients.

Furthermore, the GLM analysis to determine the main effect of target movement type showed significant interactions between the independent factors (group, type of movement, and speed [*F*_2,116_ = 14.24, *P* < 0.001]). As Figure 2(b) depicts, reaction times were slower for constant speed than during acceleration or deceleration trials. This interesting finding could point to the fact that changes in color are easier to spot when the target movement is variable, relative to when it is constant. Even if this is seemingly counterintuitive, it is consistent with our previous results when comparing the reaction time to color change in moving and stationary targets [25]. We had found that moving targets are easier and quicker to react to, possibly suggesting that following a moving target increases the attention of the subject resulting in faster responses. There was no interaction effect, however, which indicates that the two groups are affected similarly by the kinematics of the target.

### 3.2. Hit Ratios

**Figure 3:**
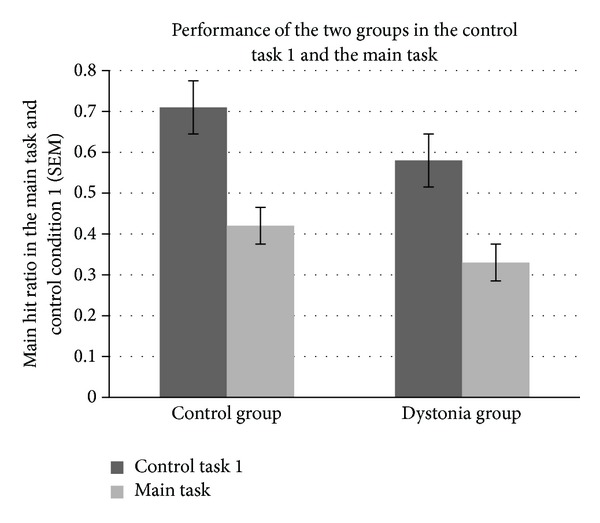
Performance in the main task and the control task 1.

The first control condition (CC1) was designed to eliminate the need of complex estimation depending on the movement types (CC1, see Figure 1). We performed a GLM analysis to compare the patient group with the control group. The dependent variable was the hit ratio, and the independent factors were the group (cervical dystonia patients and healthy controls), movement type (acceleration, deceleration, and constant), and speed (fast, medium, and slow). We observed a significant difference in the hit ratio between the healthy control group (mean = 0.71; SD = 0.04) and the patients (mean = 0.57; SD 0.18; *P* < 0.001; see Figure 3). The three-way interaction term was not significant (*F*_4,116_ = 1.60, *P* > 0.05), however, indicating that the success rate was not affected differentially by the movement type or speed of the target across groups, in contrast to the main task.

In both groups, performance in CC1 was superior to performance in the main task (*F*_1,58_ = 54.36; *P* < 0.001; see Figure 3). This is an expected result, since the main task is associated with far more complex temporal estimation of target movement parameters. Indeed, even in the control condition, patients had lower hit ratios than healthy individuals (*F*_1,58_ = 22.87; *P* < 0.001).

A comparison of performance in the first and second half of the task revealed a significant difference in hit ratios in both groups, indicating that the participants improved their performance over time (controls: *F*_1,29_ = 5.87; *P* < 0.05; patients: *F*_1,29_ = 5.27; *P* < 0.05). We observed no significant interaction between the two groups (*F*_1,58_ = 0.19; *P* > 0.05), indicating that performance changed similarly in both patients and healthy controls over time. 

**Figure 4:**
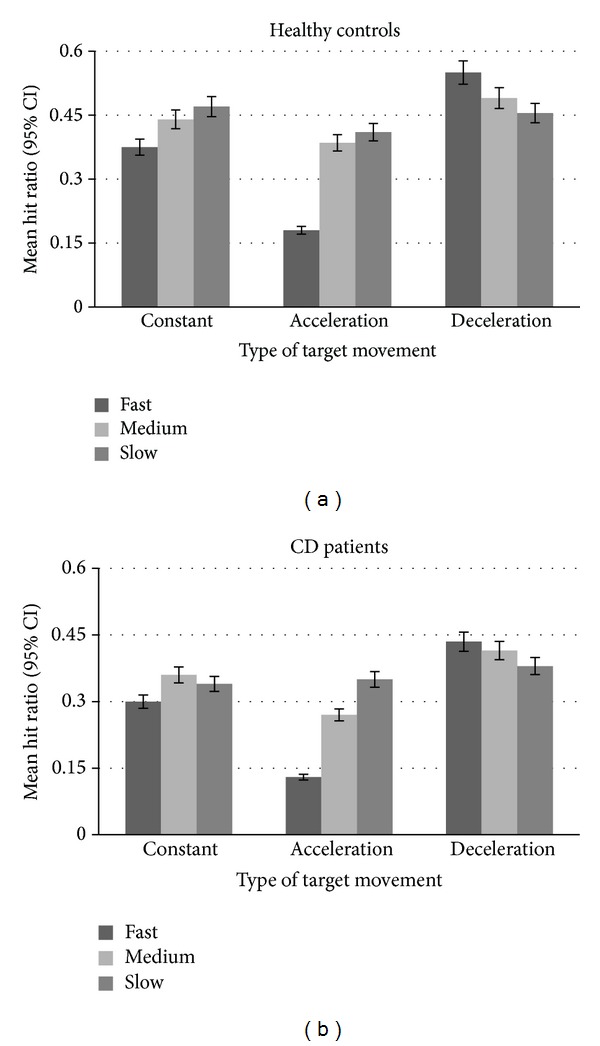
Mean hit ratio as a function of movement type and speed of the target in the main task.

Regarding the movement parameters, there was a significant effect of both movement type and speed on the hit ratio in both groups (Figure 4). Overall, the increase in target speed led to a decrease of hit ratio both in healthy (*F*_2,58_ = 15.58; *P* < 0.001) and in cervical dystonia patients group (*F*_2,58_ = 16.28; *P* < 0.001). Similarly, the deceleration and constant movements of the target lead to higher hit ratios than when the target was accelerating in both groups (*F*_2,58_ = 77.96; *P* < 0.001 for controls, and *F*_2,58_ = 77.29; *P* < 0.001 for patients). However, there was also a significant interaction between the speed and type of movement in each group (*F*_4,116_ = 54.44; *P* < 0.001 for controls, and *F*_4,116_ = 28.72; *P* < 0.001 for patients). This effect indicated that hit ratio was inversely related to speed when target moved with constant and accelerating speed, whereas this effect was reversed for targets with decelerating speeds (Figure 4).

**Figure 5:**
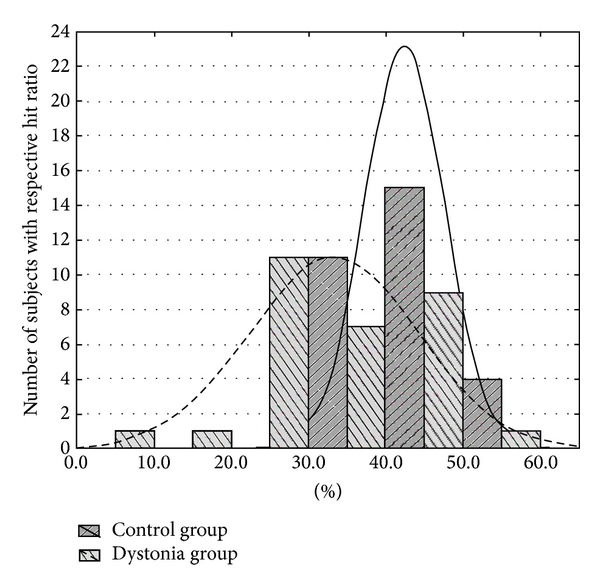
Histogram of hit ratios distributions in the patient group and the control group showing wider distribution curve for the cervical dystonia patients.

A detailed analysis of the hit rates revealed that the hit ratio distribution in the cervical dystonia group was much wider than that of the healthy controls (Figure 5). Based on this distribution, we classified the patient group into two subgroups according to a threshold set at the lower limit of the healthy group performance: Group 1 with a hit ratio comparable to the healthy controls (*n* = 15 patients), and Group 2 with lower hit ratios (*n* = 15 patients). We analyzed the individual characteristics in both patient subgroups. The parameters we focused on were age, sex, disease severity (TWSTRS score), dystonia clinical presentation (head tremor or deviation), length of the disease, and age at which the disease manifested. None of these factors differed significantly between the groups, however (*P* > 0.2). Taken together, these results imply that the presumed cerebellar deficit in cervical dystonia patients—regardless of severity—leads to worse performance in time estimation in general. In some patients, however, this ability is impaired only slightly relative to the healthy population, while in others the dysfunction manifests as a far greater “disability.”

### 3.3. Early and Late Errors

**Figure 6:**
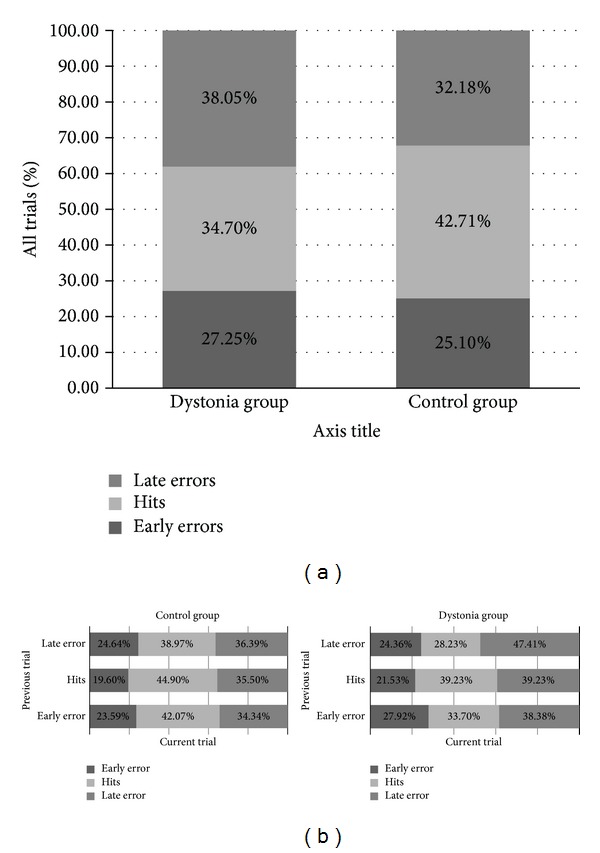
Error distribution. (a) Distribution of early and late errors in healthy subjects and cervical dystonia patients. (b) Trial-by-trial distribution of hits and errors: The effect of feedback and the impact of the success rate in the previous trial on the hit ratio and early/late errors distribution. In the graph, the error type distribution is presented as a function of the previous trial result.

We also examined the distribution of errors to determine the characteristics of unsuccessful reactions, that is, whether the subjects were too early or too late in general to intercept the target. The two types of errors seemed approximately equally distributed in the early and late spectrum in both subject groups (see Figure 6(a)); the ratio of early/late errors was 41.73/58.27% in the cervical dystonia group and 43.84/56.16% in the control group. Nonparametric tests indicate that these two groups differed in the distribution of early and late errors, however, with patients making more late than early errors (Cramer's *V* and phi coefficient = 0.21, *P* < 0.01).

### 3.4. Trial by Trial Adaptation

Figure 6(b) illustrates the distribution of early and late errors according to the performance in the previous trial. We determined whether the feedback from the previous trial had a significant impact on the performance in the current trial. We hypothesized that the subjects could benefit from the outcome of the previous trial by adjusting their motor timing in the current trial. Analyses revealed a significant effect of the preceding trial outcome on the distribution of early and late errors and hits in both the healthy control and patient group (Cramer's *V* and phi coefficient = 0.07, *P* < 0.01; and 0.08, *P* < 0.01, resp.). There were, however, slightly different qualitative outcomes when comparing healthy subjects and cervical dystonia patients. The residual standardized scores indicate that the success in the previous trial increased the hit rate on the current trial and reduced the rate of early errors in both the patient and the control group. By the same token, patients had more late errors and fewer hits in the current trial after a late error in the preceding trial, whereas for the healthy group late errors did not lead to a significant change in hit ratio. 

### 3.5. Hit Ratio and the Time Window

**Figure 7:**
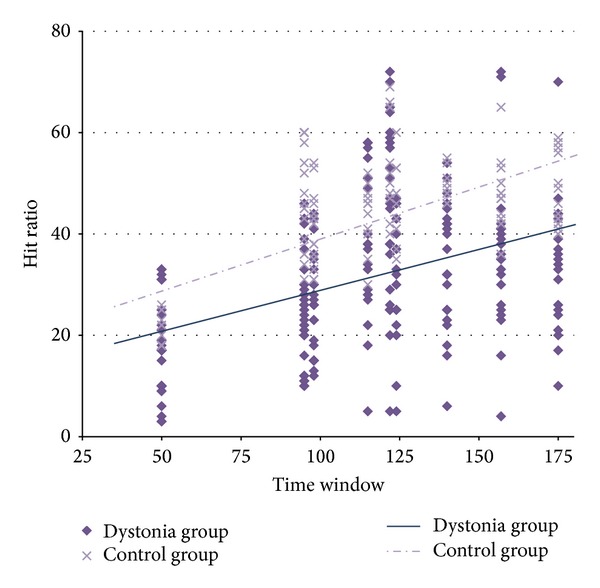
The effect of response window width and hit ratio. The relationship between hit ratio (percentage) and window width (milliseconds) for the two groups.

As previously mentioned, the temporal window within which a successful outcome is possible is an indication of the task difficulty; the longer this time interval, the greater the likelihood that subjects could execute the movement successfully. We computed the correlation between window width and hit ratio for both the healthy control and patient group and then compared these correlation coefficients between them.  Figure 7 illustrates the relationship between hit ratio (percentage) and window width (in milliseconds) for both groups. While we obtained a significantly positive correlation coefficient for both groups (a higher hit ratio corresponded to wider window width), we did not find any significant difference between them. In fact, the hit ratios of two groups were affected similarly by the window width indicating that the slopes were similar (*F*_1,536_ = 0.411; *P* > 0.05; ~20% change in hit ratio for 100 ms window width in each group). Also, there was greater variability among patients: while window width explained 31.7% of the variability in hit ratio among healthy individuals, this factor explained only 19.3% of the variability among patients. This finding may be related to the heterogeneity of the disease manifestation among patients.

## 4. Discussion

This current study investigated whether patients with cervical dystonia exhibit impaired performance on a motor-timing task known to require cerebellar input. Using a motor-timing computer task, we reveal the following pattern of results: the principal finding is a lower hit ratio of the patients in comparison to the control group in a specific motor-timing task in association with significantly slower reaction time to a simple color change in the dystonia group. On the other hand, the ability to take advantage of the wider time window was comparable in cervical dystonia patients and the control group. Likewise, there was a significant effect of both movement type and speed on the success rate in the control group and cervical dystonia patients not showing a prominent difference between those two groups. Visual-motor integration at the millisecond range, which is believed to be a substantial constituent of cerebellum responsibilities [28, 30, 31], is of crucial importance for success on this task. Although the cerebellum is not noted traditionally as one of the major sources of dystonia development, interest towards this neuronal structure has increased recently [8, 9, 38], with its role in the pathophysiology of the dystonia suggested by animal models [7, 39, 40], imaging studies [11, 41, 42], neurophysiological studies [10], and even secondary cervical dystonia analyses [18, 43]. Our findings demonstrate that the temporal estimation of target parameters and requirements for a quick motor response posed a fundamental problem for the cervical dystonia patients. This is consistent with the notion that the cerebellum may also be affected by this disease.

It remains to be determined whether the cerebellum stands at the very pathophysiology origin of cervical dystonia or it forms only a component of a complex network compensating for dysfunction of other parts of the brain. Nevertheless, our data indicate that cervical dystonia subjects show decreased performance on a task in which the cerebellum has been shown previously to play an essential role [23–25]. Our results are also consistent with the current theories of a discrete event timing network responsible for time interpretation and assessment in the millisecond range, located in cerebellum [28, 30, 31]. The cerebellum is hypothesized to be a major node in timing tasks with noncontinuous movements [44, 45], a function fitting undeniably with the spirit of our task. The cerebellum hosts a vast convergence of mossy fiber inputs [46], and it is known to be involved in the synthesis of information from virtually all brain areas. As such, the cerebellar structure is well situated for complex predictions of an integrative character, providing crucial data for further processing by the cerebral cortex [47, 48].

Of course, the nature of our study makes it impossible to assess complexly the extent of eventual cerebellar dysfunction; this network node is associated with a wide spectrum of functions of which we were interested specifically in only one, that is, movement timing. Performance on other tasks involving cerebellum function, such as attention [19, 20], associative learning [21], and motivation control [22], remains to be investigated. This leaves a window for further research into the association between the cerebellum and dystonia open. In particular, imaging studies of cerebellum connectivity and its eventual functional abnormities in cervical dystonia will be of great importance. Nevertheless, our observation of decreased performance in a timing task in patients with no clear neurological cerebellar signs (e.g. ataxia and dysmetria) could be attributed to abnormal cerebellar activity in dystonia—specifically a gain of cerebellar function similar to epilepsy in the cerebral cortex [39, 49]. This very abnormity might be related closely to cortical excitability disorder in cervical dystonia patients [50], leading possibly to typical cervical dystonia symptoms. 

Of course, it would be inappropriate to dismiss completely the contribution from the basal ganglia, which is both an important pathophysiological node with direct connection to dystonia in general and an essential contributor to motor timing. In our previous study, however, dysfunction of the basal ganglia, a structure noted particularly in attention and memory tasks in the range of seconds [29, 32, 51], did not lead to lower performance in our motor-timing task [25]. Patients with Parkinson's disease, despite their hypokinesia, performed at levels comparable to healthy subjects. In contrast, patients with essential tremor and spinocerebellar ataxia, disorders connected with cerebellum dysfunctions, showed comparable problems in time estimation and movement analysis. Moreover, the late error ratio in spinocerebellar ataxia patients was increased in comparison with healthy controls—a trend observed also in cervical dystonia patients. Another important observation in our study is that the cervical dystonia patients and the spinocerebellar ataxia patients, in addition to poor performance, had abnormal timing adjustment. 

Furthermore, we should not overlook the possibility that the impaired performance in cervical dystonia patients was caused by oculomotor problems. Should this be true, the distribution of errors should shift in favor of late errors in the cervical dystonia patients. This was not the case, however. Moreover, the increasing speed and acceleration/deceleration posing higher demands for the visuomotor abilities of the subject, that is, the shorter time window, influenced cervical dystonia patients to the same extent as healthy controls. We also observed that the reaction time was not affected by the parameters of the moving target (i.e. speed or acceleration) differently between the two groups. This rejects further the possibility that differences between the groups arise from oculomotor difficulties in the cervical dystonia group.

Lastly, the slight abnormal shoulder posture of some cervical dystonia subjects might be considered to contribute to our pattern of findings. We find this unlikely, however, as the task is designed specifically to minimize the aspect of unnatural posture. Subjects did not use the whole limb or bigger limb segments to intercept the target; they were required merely to push the response button with their finger.

Our results contribute to our understanding of both the role of the cerebellum dystonia pathophysiology and its function in general. A growing body of research in this former area implicates an increasing number of defective neural network nodes in this disease [52, 53], thus challenging the traditional view of dystonia. Indeed, although it is clear that basal ganglia play a significant role in dystonia pathophysiology, our findings pointing to cerebellum dysfunction, together with the results of clinical and animal studies [39, 54, 55], associate dystonia with defective interactions among different components of the motor network rather than the dysfunction of any one node. Standing on the doorstep of the first real complex insight and understanding of cerebellum and the central nervous system itself, backed up by ever-advancing technological possibilities, we cannot afford to overlook this slowly crystallizing role of the cerebellum in the pathophysiology of cervical dystonia.

## Figures and Tables

**Table 1:** Further information about the cervical dystonia patients.

No.	Demographics		Time since onset (years)	Tremor	Dominant head position distortion	TWSTRS	Treatment	Success rate
	Age	Sex						
1	61	F	10		Torticollis	15	Botulotoxin	34.88%
2	35	M	7	Yes	Torticollis	5	Botulotoxin	42.28%
3	65	F	15		Laterocollis	4	Botulotoxin	25.62%
4	63	F	9		Torticollis	7	Botulotoxin	13.40%
5	73	F	32		Laterocollis	10	Botulotoxin	33.64%
6	71	M	27		Laterocollis	8	Botulotoxin	22.69%
7	63	M	38	Yes	Torticollis	18	Botulotoxin	31.33%
8	51	F	4	Yes	Torticollis	3	Botulotoxin	43.06%
9	54	F	7	Yes	Laterocollis	14	Botulotoxin	35.96%
10	23	M	6		Torticollis	5	Botulotoxin	35.96%
11	38	F	12	Yes	Torticollis	13	Botulotoxin	45.06%
12	45	M	15		Retrocollis	10	Botulotoxin	43.52%
13	60	M	27		Laterocollis	15	Botulotoxin	14.61%
14	48	M	22		Torticollis	13	Botulotoxin	21.14%
15	49	M	13		Torticollis	8	Botulotoxin	42.28%
16	33	M	18	Yes	Laterocollis	18	Botulotoxin	24.38%
17	59	F	13		Laterocollis	7	Botulotoxin	24.54%
18	59	M	16		Torticollis	11	Botulotoxin	46.60%
19	62	F	13		Torticollis	7	Botulotoxin	29.94%
20	41	F	7	Yes	Torticollis	10	Botulotoxin	39.81%
21	49	M	11	Yes	Laterocollis	15	Botulotoxin	27.93%
22	50	M	8		Torticollis	6	Botulotoxin	28.86%
23	58	M	6		Laterocollis	11	Botulotoxin	41.67%
24	60	F	12	Yes	Torticollis	18	Botulotoxin	51.85%
25	32	F	12	Yes	Torticollis	5	Botulotoxin	29.01%
26	66	M	6		Laterocollis	4	Botulotoxin	40.43%
27	21	F	3	Yes	Torticollis	18	Botulotoxin	49.23%
28	68	F	4		Torticollis	10	Botulotoxin	24.85%
29	62	F	13	Yes	Torticollis	13	Botulotoxin	33.18%
30	41	F	4		Laterocollis	5	Botulotoxin	25.77%
